# Editorial: Data integration and physiological modelling

**DOI:** 10.3389/fphys.2022.1116488

**Published:** 2023-01-04

**Authors:** Yasha Hasija

**Affiliations:** Department of Biotechnology, Delhi Technological University, Delhi, India

**Keywords:** data integration, physiological modelling, computational modelling, OMICS data, biological big data

## 1 Introduction

The main objective of physiological research is to gain a comprehensive understanding of how living systems work. Therefore, since its infancy, physiology has endeavoured to connect multiple levels of biological organization. Mathematical and computational methods are increasingly being used to model a small number of components at multiple levels of the biological organisation hierarchy. The connection between molecules and phenotypes is made possible by quantitative modelling of physiological processes. Ample amount of data required for the development of physiological models, since models with insufficient data may fail to approximate the real-world scenario. In the 1960s and 1970s, the first integrative models of human physiology were created. Computational approaches have been used to investigate and characterise physiological reactions since William Harvey calculated cardiac output and ejection fraction in 1628 (Keele, 1957). Quantitative Circulatory Physiology (QCP) is a computer programme (software package) that simulates multiple organ systems in the human body and was launched in 1972 (Coleman and Randall, 1983).

Integrating high-throughput data from genomics, transcriptomics, proteomics, and metabolomics into a significant, large dataset is necessary. Even though there is a lot of data being collected in the biological field, it is still hard to understand how a system works as a whole because there aren’t many ways to put all the data together.

The five publications that make up this Research Topic cover a variety of data integration and physiological modelling studies. The contributions of authors from various backgrounds who have conducted empirical studies, developed computational models, and written reviews have been helpful for this Research Topic. In this editorial, we provide an overview of these interesting articles by categorising them based on their conceptual design.

## 2 Data integration and modeling in early diagnosis of diseases

Predictive physiological models and simulations that combine physiological knowledge with clinical data-derived patient information have enormous potential to improve our understanding of disease progression and treatment. They have the potential to improve patient selection and optimise interventions for a wide range of disorders. Currently, a large proportion of this potential is untapped. When treated promptly, shock, a leading cause of death in intensive care units, is reversible. In this issue’s study, Vats et al. developed a deep learning-based model for continuous hemodynamic shock monitoring. The model is fed with non-contact thermal imaging time-series data and patient heart rates to forecast the shock state for the next 6 h. Another study by Singh et al. used machine learning (ML) models trained on physiological vitals to predict hypothermia 30 min to 4 h before it is occurrence. The most serious concern in intensive care units (ICUs) is hypothermia, a potentially fatal condition that occurs when the body temperature falls below 35°C. Such processes can now be monitored efficiently thanks to optical sensors and AI algorithms.

Kaselimi et al. cover imaging techniques and optical sensors in a review article. The study explores sensor properties as well as patient physiology. Many techniques use visible and infrared bands (in varying ranges) and AI tools for various monitoring strategies, and the AI tools used vary depending on the sensor type. This extensive review of the literature, which includes summaries of numerous recent scientific articles, covers the methods for AI-assisted DFU (Diabetic Foot Ulcer) monitoring. The article also highlights these strategies and discusses the challenges of incorporating them into a practical and dependable framework for patient remote management.

## 3 Data integration and modeling in therapeutic targets

In the past 10 years, the field of “systems biology” has concentrated on the fusion of biology, medicine, and mathematics. The present challenge is to develop focused therapeutic techniques based on recent discoveries in genomics and proteomics. The majority of systems biology methodologies that integrate several omics technologies, whether explicitly or implicitly, ranging from statistical inference methods to mechanistic modelling, are based on network analysis (Oulas et al., 2019; Yue and Dutta, 2022).

The integration of knowledge and data from several sources may be necessary for successful drug design, drug repurposing, and multi-target therapy. Biological networks are an effective tool for understanding these connections and simulating the processes behind the effectiveness of different treatments. Numerous neurodegenerative diseases are linked to disruptions in the sphingomyelin signalling system. TNF is a strong cytokine that promotes inflammation and is produced in response to several neurological diseases, including Parkinson’s and Alzheimer’s. A detailed examination of the system dynamics was made by computational modelling and simulation of the inflammatory pathway. The results of the study conducted by Banaras et al. found sphingolipids (S1P) and a number of caspases (CASP2, CASP8, and CASP9) as powerful proteins with therapeutic importance in later stages of the disease. Furthermore, the drug dosage study assessed the efficacy of three pharmaceuticals on the level of neuroinflammatory reactions, namely Etanercept, Nivocasan, and Scyphostatin.

## 4 Textual data integration and modeling

The majority of scientific communication in biomedicine is still text-based. Over the past few years, text mining methods for the automatic extraction of valuable biological information from unstructured text that may be used directly for systems biology modelling have significantly advanced. Over the past 15 years, technologies have been created to automatically identify and extract important biomedical information from unstructured text. Text-mining technology has grown rapidly from locating and retrieving PubMed abstracts to extracting complicated biological context from full-text sources (Fluck and Hofmann-Apitius, 2014; Wang et al., 2022).

Ahmed et al. demonstrated the possibility of large-scale text mining for gaining unique insights, with an emphasis on ‘microbiome’. In the beginning, they made an effort to comprehend the geographic distribution of microbiome research based just on data from abstracts (text + metadata). Subsequently, they focused on discovering connections and interactions between three important categories in the microbiome space: disease terms, dietary terms, and microbes.

## 5 Conclusion

Most of the publications in the current Research Topic have focused on creating computational models for the early identification of diseases and the discovery of therapeutics. We anticipate that many of these multi-dimensional modalities will soon be measured in a single patient, allowing information from several modalities to be integrated to provide a detailed description of a specific condition or spectrum of disorders. We can learn more about the operation and mechanism of a physiological process by creating models. It is safe to expect that every quantitative model will eventually fall short because biology is recognised as the science of exceptions. The inadequacy of these models prompts us to look into what was lacking, and in the course of that investigation, a few new components, pathways, molecules, and so forth are integrated to increase their accuracy. New findings, component interactions, and a deeper comprehension of an organism’s, organ’s, or system’s typical functional processes are made, as this iterative process goes on. The development of computational physiological models and the integration of various omics datasets can thus aid in our understanding of system biology and its homeostasis.
